# Technical efficiency and its convergence among village clinics in rural China: evidence from Shanxi Province

**DOI:** 10.3389/fpubh.2024.1364973

**Published:** 2024-05-10

**Authors:** Yun Ye, Richard Evans, Xiaojun Huang, Wei Xu, Wei Lu

**Affiliations:** ^1^School of Management, Hainan Medical University, Haikou, Hainan, China; ^2^Faculty of Computer Science, Dalhousie University, Halifax, NS, Canada; ^3^PBC School of Finance, Tsinghua University, Beijing, China

**Keywords:** village clinics, technical efficiency, convergence, PGS, BA, RTETI

## Abstract

**Introduction:**

Village clinics (VCs) are the foundation of the three-tiered health service system in China, delivering basic and routine outpatient services to citizens in rural China. VC technical efficiency and its convergence play a critical role in policy decisions regarding the distribution of health service resources in rural China.

**Methods:**

This study measured VC technical efficiency (using the slacks-based measure model), its convergence (using the convergence model), and the factors that influence the convergence in Shanxi Province, China. Data were obtained from the *Shanxi Rural Health Institute 2014–2018 Health Statistics Report*, which involved 3,543 VCs.

**Results:**

The results showed that VC technical efficiency was low and differed by region. There was no α convergence in VC technical efficiency, but evidence of β convergence was found in Shanxi. The main factors that influence convergence were the building area of each VC (BA), proportion of government subsidies (PGS), and ratio of total expenditure to total income of each VC (RTETI).

**Conclusion:**

The government should increase investments in VCs and improve VC technical efficiency. Meanwhile, the government should be aware of and take measures to curb the inequity in VC technical efficiency among different regions and take suitable measures to curb this disparity.

## 1 Introduction

China's three-tiered rural health service system comprises three types of primary healthcare institutions (PHCIs): village clinics (VCs), township health centers, and county hospitals. VCs serve as the foundation of the three-tiered health service system, delivering basic and routine outpatient services to local villagers and undertaking important responsibilities for citizens residing in rural areas (1). According to the statistics of the National Health Commission of the People's Republic of China (2), the number of visits to VCs was >1.3 billion in 2021, accounting for >31% of visits to PHCIs.

VC technical efficiency represents VCs' ability to transform health inputs into outputs. Since China's healthcare reform in 2009, the Chinese government has increased health resource investments in rural areas to provide high-quality healthcare for rural citizens, including improving VC technical efficiency (3). However, due to differences in economic development, VC technical efficiency considerably varies among regions (4). This disparity in the efficiency levels of VCs indicates an imbalanced distribution of health resources among VCs (5). Policymakers need to understand the VC technical efficiency level and assess whether there has been progress in reducing the inequity of VC technical efficiency in rural areas over time.

The convergence of VC technical efficiency can be used to assess the changing tendency of inequity of resource allocation applied to the healthcare field (6–8). Undoubtedly, the convergence of VC technical efficiency plays a critical role in health resource distribution for policymakers in China. First, if VC technical efficiency is found to converge to a steady-state level, policymakers can formulate a comprehensive medical and health resource policy that is applicable across the field. Conversely, if convergence is not achieved, a set of distinct policies can be formulated to ensure convergence. Second, establishing health resource policies that help VC technical efficiency converge to a steady-state level improves not only health resource allocation equity but also health resource utilization efficiency in China's rural areas. In addition, it is crucial for the government to understand how to intervene in the convergence in order to implement precise measures to narrow the VC technical efficiency differences between rural areas in China.

For the abovementioned purposes, this study measured VC technical efficiency, the convergence of VC technical efficiency, and the factors that affect convergence. Overall, this study answered the following critical questions: (1) What is the level of VC technical efficiency in rural areas of China? (2) Is VC technical efficiency converging? and (3) What factors influence the convergence of VC technical efficiency?

The structure of the article is as follows. Section 2 provides an overview of the relevant research literature. Section 3 introduces the methods in detail. Section 4 presents the research results. Section 5 reports the discussion. Section 6 presents the conclusion and limitations of the study.

## 2 Literature review

An increasing number of studies have been conducted on the efficiency of rural health institutions (9–13). The efficiency of rural health institutions in China is garnering increased research attention, with the majority focusing on hospitals in counties and towns. For instance, Zhong et al. estimated the efficiency of PHCIs and its influencing factors across 86 counties in Hunan Province. They found that, although the quantity of health resources in PHCIs in Hunan Province has increased significantly, PHCIs remain inefficient in most counties (14). Similarly, Zheng et al. analyzed the efficiency of China's rural township-level medical service systems during 2013–2017 and found that 11 out of 27 provinces were inefficient and 10 out of 27 provinces had lower efficiency than the average scores (4). Similarly, Zhang et al. measured the efficiency and productivity of primary healthcare resource allocation in China and found that >80% of the provinces had inefficient PHCIs and the productivity of the PHCIs declined by 0.6% from 2012 to 2016 (15).

Although several studies have explored VCs, they mainly focused on VC medical waste management and VC utilization and its determinants. For instance, Gao et al. analyzed VC medical waste management based on survey data and found that the average rate of medical waste generation in the sampled VCs was ~0.65 kg/day or 0.17 kg/patient/day, and the total quantity of medical waste generated was noteworthy (16). Chen et al. estimated that the probability that individuals sought care at VCs when ill decreased by 44% between 2011 and 2018, whereas the utilization of outpatient services in county hospitals increased by 56% and patient self-treatment increased by 20% (1). Bark et al. found that VC utilization can be explained by economic status and walking time to VCs (17).

Convergence was initially used to analyze economic growth and gaps among countries or regions (18, 19), and later, it was used by a small number of studies to analyze factors related to healthcare systems. Gächter et al. analyzed the convergence of age-standardized mortality (as an indicator of health status) and found evidence of absolute and conditional β-convergence but no α-convergence (20). Traoré examined the convergence of public health expenditure among sub-Saharan African countries and found no evidence of convergence (21). Zhang et al. assessed the convergence of China's regional government health expenditure and found evidence of α convergence and β convergence (8). Shen et al. analyzed the convergence of healthcare resource supply in the Yangtze River Delta region in China and revealed that it increased significantly and converged rapidly (22). Kasman et al. investigated whether convergence existed in technical efficiency and productivity levels of the healthcare systems of 26 EU members and an EU candidate country; they found evidence of both α convergence and β convergence (23).

The literature review indicates that studies generally focus only on assessing the efficiency of hospitals in counties and towns. The issue of convergence has been examined mostly in the context of health expenditures and health outcomes. Unlike the existing literature, this study conducted a convergence analysis to identify convergence of technical efficiency among VCs and determined the factors that affect convergence. This finding provides a basis to optimize the dynamic management of rural health resources to promote rational health resource allocation.

## 3 Methods

### 3.1 Study area and data collection

Shanxi Province, located in central China, has a population of 34.81 million and had a gross domestic product per capita of RMB¥73,675 in 2022 (24). Shanxi has 26,355 VCs, ranking 7th among 31 provinces/municipalities in China in terms of the number of VCs. These VCs provide medical services to 12.73 million rural residents in Shanxi (36.6% of the population) every year (2).

**Figure 1:**
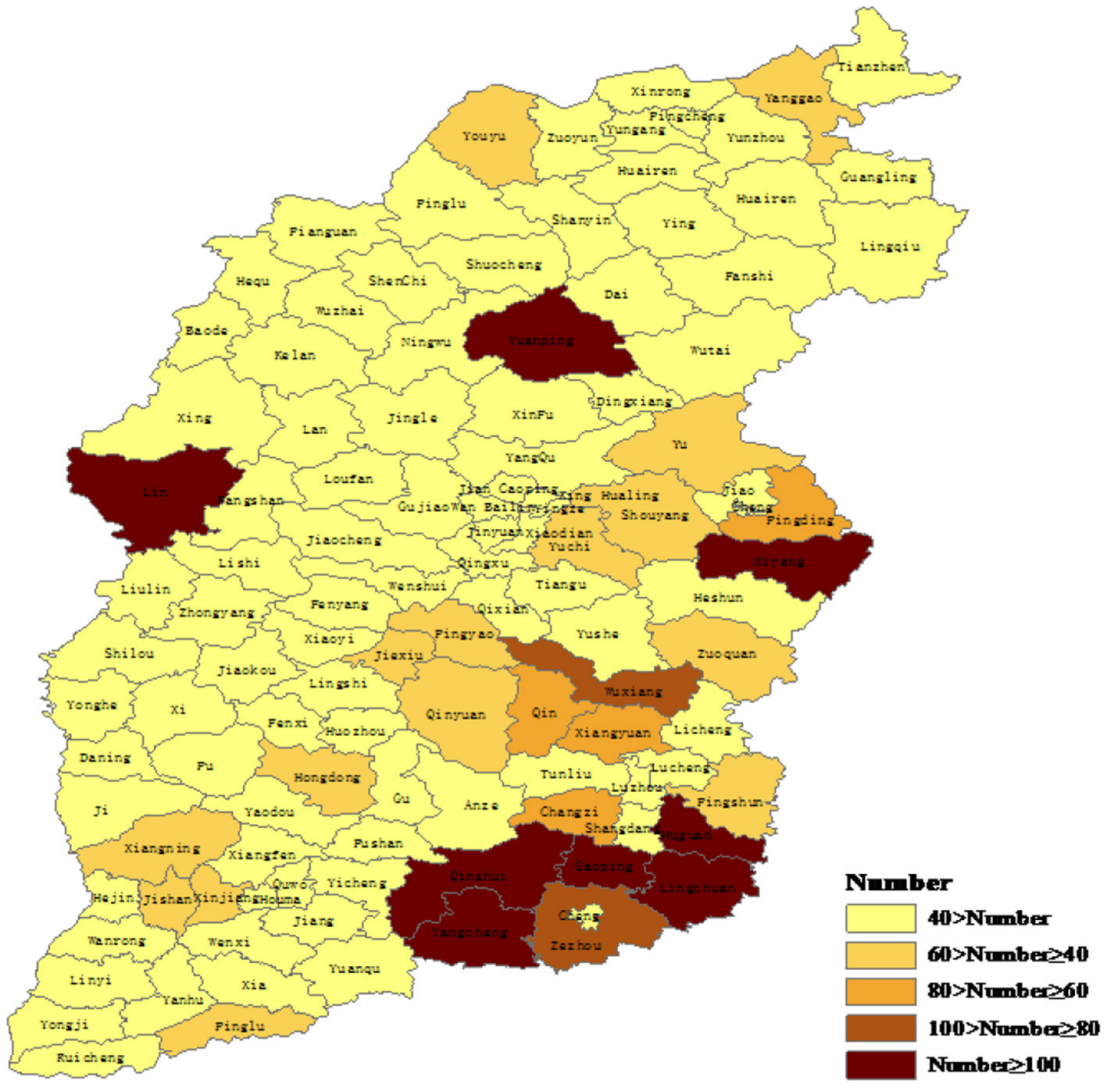
Number of village clinics (VCs) sampled in each county of Shanxi Province.

Data were collected from the Shanxi Rural Health Institute 2014–2018 Health Statistics Report (25), and 3,543 VCs were analyzed (13.44% of VCs in Shanxi). These VCs were selected from 100 counties (including prefecture-level cities and municipal districts) in 11 cities of Shanxi (Figure 1).

### 3.2 Technical efficiency analysis

Data envelopment analysis (DEA), which can be used to measure VC technical efficiency, has been widely used in previous research (26–33). However, conventional radial DEA models, such as the Constant Returns to Scale Ratio Model (CCR model) proposed by Charnes, Cooper, and Rhodes) and Variable Returns to Scale Model (BCC model) proposed by Banker, Charnes, and Cooper), only include the proportion of all inputs or outputs reduced or increased. When measuring an institution's efficiency, the effect of slack variables is not considered, which may result in the deviation of the efficiency estimate from the ground truth. Tone proposed a slacks-based measure (SBM) model (as a function of the farthest distance to the efficiency frontier) that solved the problem of radial DEA models not including slack variables (34, 35). The present study adopted the following SBM model (Equation 1):


(1) $$\begin{array}{l} \text{To objective function}:\text{min}\rho =\frac{1-\frac{1}{m}\overset{m}{\underset{i=1}{\sum }}\frac{s_{i}^{-}}{x_{ik}}}{1+\frac{1}{q}\overset{q}{\underset{r=1}{\sum }}\frac{s_{r}^{+}}{y_{rk}}} \end{array}$$

(2) $$\begin{array}{l} \text{Constraints}:x_{k}=x\lambda +s^{-},\;y_{k}=y\lambda -s^{+},\lambda ,s^{-},s^{+}\geq 0 \end{array}$$

where *m* and *q* are inputs and outputs, respectively, of each VC, *x*_*ik*_ *andy*_*rk*_ are the *ith* input and the *rth* output, respectively, of the VC, *s*^−^ and *s*^+^ are the slack variables of the *ith* input and *rth* output, respectively, and ${s_{i}}^{-}/x_{ik}$ and ${s_{i}}^{+}/x_{ik}$ are the inefficiencies of the *ith* input and *rth* output, respectively. Regarding the constraints (Equation 2), *x*_*k*_ and *y*_*k*_ are the input and output, respectively, of the *kth* VC, λ is the adjustment matrix, *xλ* and *yλ* are the input and output, respectively, of the frontier production line, *s*^−^ *and* *s*^+^ are the slack variables of the input and output, respectively, and ρ lies between 0 and 1. If ρ = 1, the VC is highly effective and located at the efficiency frontier, and each slack is 0. If is close to 0, the VC is inefficient.

Unlike town and county hospitals, VCs mainly provide rural citizens with general diagnosis and treatment, along with referrals for common diseases. Therefore, VC input and output indicators are considerably different from those of county and town hospitals. We selected input and output variables based on the input and output variables in the literature (36–38), combined with the characteristics of VCs. The input variables were the number of rural doctors, the number of rural doctors who underwent technical training, the quantity of medical equipment, and drug expenditure (Table 1). The output variables were the number of patients who visited and income from essential drugs (Table 1). The number of rural doctors who underwent technical training was chosen as an input variable because the increased training can improve doctors' medical service capabilities and promote their efficiency. *Management Measures of VCs (Trial Implementation)*, issued by the National Health Commission of the People's Republic of China, clearly proposed to establish a VC training system and encourage doctors employed in VCs to receive further medical education (39).

**Table 1:** Input and output variables of a technical efficiency measurement model.

**Category**	**Variable**	**Definition**	**Unit**
Inputs	Number of rural doctors	Number of rural doctors employed in each VC	Person
	Number of rural doctors who underwent technician training	Number of rural doctors that participated in the technician training program organized by the government in each VC	Person
	Quantity of medical equipment	Total quantity of examination beds, refrigerators, Chinese medicine cabinets, western medicine cabinets, disposal tables, computers, and other medical equipment in each VC.	/
	Drug expenditure	Expenditure of each VC on drugs listed in the National Essential Medicines List	Yuan
Outputs	Number of patients who visited	Number of patients who visited each VC	Person
	Income from essential drugs	Income obtained by each VC from the sale of drugs listed in the National Essential Medicines List	Yuan

### 3.3 Convergence analysis

Convergence analysis is used to identify whether there is inequity of VC technical efficiency among different VCs and the factors that affect the convergence. There are two types of convergence analysis: α convergence and β convergence (40).

#### 3.3.1 α convergence

α convergence refers to the process whereby the VC technical efficiency changes over time. α convergence (Equation 3) was calculated based on the coefficient of variation (CV) of VC technical efficiency, as follows (41).


(3) $$\begin{array}{l} CV=\sqrt{\frac{1}{n}{\overset{n}{\underset{i=1}{\sum }}\left(TE_{i}-\overset{\text{\_}}{TE_{i}}\right)}^{2}} \end{array}$$

where *TE*_*i*_ is the technical efficiency of the *ith* VC, and *n* is the total number of VCs.

If CV decreases over time, the efficiency exhibits α convergence, that is, the disparity in technical efficiency among VCs narrows. By contrast, if CV increases over time, the disparity in technical efficiency among VCs expands.

#### 3.3.2 β convergence

β convergence can be divided into absolute β convergence and conditional β convergence. Absolute β convergence indicates that the lower the initial VC technical efficiency, the higher the average annual growth rate of the efficiency of that VC. Therefore, with the passage of time, the inefficient VCs will gradually reach the same level as the more efficient VCs, resulting in the convergence of the efficiency of VCs to a steady-state level. Conditional β convergence considers the effect of other factors on the average annual growth rate of efficiency. Absolute β convergence (Equation 4) and conditional β convergence (Equation 5) were calculated as follows (42):


(4) $$\begin{array}{l} \ln\;\frac{TE_{i,t+1}}{TE_{i,t}}=\alpha +\beta \ln\;TE_{i,t}+\varepsilon_{i,t} \end{array}$$

(5) $$\begin{array}{l} \ln\;\frac{TE_{i,t+1}}{TE_{i,t}}=\alpha +\beta \ln\;TE_{i,t}+\gamma X_{i,t}+\varepsilon_{i,t}, \end{array}$$

where *TE*_*i, t*+1_ and *TE*_*i, t*_ are the technical efficiencies of the ith VC in the periods *t*+1 and *t*, respectively, ln (*TE*_*i, t*+1_/*TE*_*i, t*_) is the average annual growth rate of technical efficiency of the *ith* VC, *X*_*i, t*_ are other variables that affect ln (*TE*_*i, t*+1_/*TE*_*i, t*_), and are the error terms.

A significant and negative β coefficient indicates that the average annual growth rate of the technical efficiency is negatively correlated with the efficiency score in the base period, signifying a “catch-up effect” among VCs. In other words, the VCs with a low initial technical efficiency score have a higher growth rate, and the technical efficiency of all VCs will eventually approach the steady-state level. By contrast, a significant and positive β coefficient indicates a widening disparity in technical efficiency among VCs.

In the conditional β convergence model, the following variables that affect the average annual growth rate of the technical efficiency were considered: the number of people served (PS), building area (BA), proportion of government subsidies (PGS), and ratio of total expenditure to total income (RTETI; Table 2) (43).

**Table 2:** Influencing variables used in convergence analysis of village clinics (VCs).

**Variable**	**Definition**	**Unit**
Number of people served (PS)	Number of people served by each VC	Person
Building area (BA)	Building area of each VC	Sm^2^
Proportion of government subsidies (PGS)	Subsidies from the government as a proportion of the total income in each VC	%
Ratio of total expenditure to total income (RTETI)	Total expenditure of each VC divided by the total income of each VC	%

## 4 Results

### 4.1 VC technical efficiency

**Figure 2:**
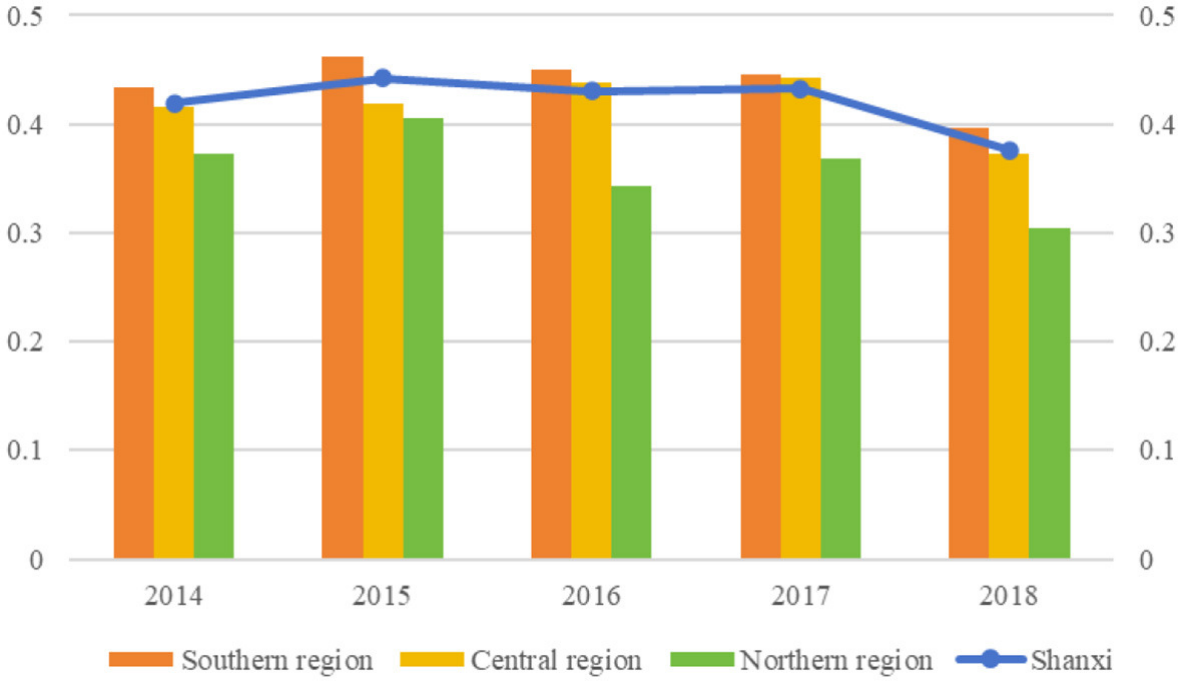
Village clinic (VC) technical efficiency scores in Shanxi from 2014 to 2018.

Figure 2 shows the VC technical efficiency scores in Shanxi Province during 2014–2018. VC technical efficiency generally showed a fluctuating downward trend, from 0.4196 in 2014 to 0.3764 in 2018. The average scores during 2014–2018 were 0.4196, 0.4423, 0.4304, 0.4325, and 0.3764, respectively, and 57.8% of VCs had efficiency lower than the average score in 2018. In addition, most of the VCs were inefficient (scores < 1), with 3,424 (96.6%), 3,414 (96.4%), 3,432 (96.9%), 3,418 (96.8%), and 3,435 (97.0%) inefficient VCs in 2014–2018, respectively.

As for the technical efficiency of VCs of different regions, the average scores of the southern, central, and northern regions were 0.4376, 0.4176, and 0.3584, respectively. The southern region had the highest average score during 2014–2018, while the northern region had the lowest average score, indicating the largest technical efficiency disparity between the southern and northern regions (0.0792). VC technical efficiency in the southern, central, and northern regions also showed a fluctuating downward trend, from 0.4336, 0.4161, and 0.3722, respectively, in 2014 to 0.3970, 0.3732, and 0.3036, respectively, in 2018. The northern region exhibited the largest decline (18.42%).

### 4.2 Convergence analyses

#### 4.2.1 α convergence

**Figure 3:**
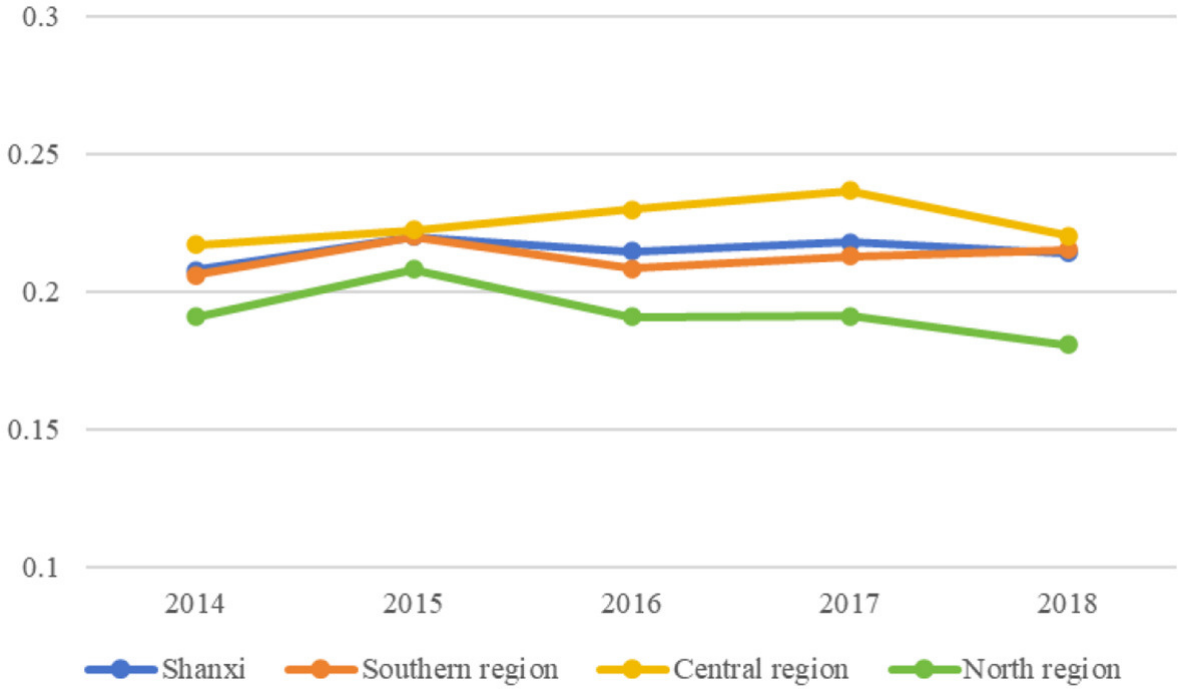
α convergence of village clinic (VC) technical efficiency in Shanxi from 2014 to 2018.

The convergence analysis depicted in Figure 3 demonstrated that the CVs of the VC technical efficiency were 0.2080, 0.2203, 0.2149, 0.2181, and 0.2142 for 2014–2018, respectively. For Shanxi VCs, the CVs exhibited an upward trend from 2014 to 2018, indicating an overall divergence trend. However, the trend was unstable; the CV increased from 2014 to 2015, decreased from 2015 to 2016, increased slightly from 2016 to 2017, and finally decreased from 2017 to 2018.

CVs in the southern and central regions exhibited an overall upward trend from 2014 to 2018, whereas those in the northern region exhibited an overall upward trend from 2014 to 2017 with a slight decline in 2018. These trends indicate the general divergence of VC technical efficiency in these three regions. However, the degree of divergence differed among regions, with the southern and central regions showing a greater degree of divergence than the northern region.

#### 4.2.2 β convergence

A fixed-effect model was adopted for β convergence analysis because the result of Hausman test showed that the statistics for the VCs in Shanxi and the three regions (i.e., southern, central, and northern) were all significant at the 1% level.

Table 3 displays the absolute and conditional β convergence coefficients. β convergence coefficients of the VCs in Shanxi and the three regions were negative and significant, indicating the existence of absolute β convergence of VC technical efficiency, that is, the average annual growth rate of efficiency was negatively correlated with the efficiency score in the base period. This finding suggests that the underdeveloped regions (with a low initial technical efficiency) were catching up with the advanced regions, gradually narrowing the efficiency gap between VCs among the regions. The VC technical efficiency in Shanxi (from the provincial perspective) and that in the three regions (from the regional perspective) tended to converge to a steady-state level.

**Table 3:** β convergence (absolute and conditional) of village clinic (VC) technical efficiency and the influencing factors.

	**Shanxi**		**Southern region**		**Central region**		**Northern region**	
	**Absolute**β **convergence**	**Conditional** β **convergence**	**Absolute** β **convergence**	**Conditional** β **convergence**	**Absolute** β **convergence**	**Conditional** β **convergence**	**Absolute** β **convergence**	**Conditional** β **convergence**
*TE*_*i, t*_	−0.9823^***^	−0.9829^***^	−0.9778^***^	−0.9800^***^	−0.9296^***^	−0.9322^***^	−1.0846^***^	−1.0821^***^
	(0.0132)	(0.0127)	(0.0184)	(0.0181)	(0.0240)	(0.0216)	(0.0268)	(0.0264)
PS		−0.0013		0.0035		−0.0039		−0.0002
	(0.0044)		(0.0088)		(0.0060)		(0.0084)
BA		0.0564^***^		0.0556^***^		0.0776^***^		−0.0529
	(0.0138)		(0.0204)		(0.0228)		(0.0324)
PGS		−0.2553^***^		−0.2054^***^		−0.4308^***^		−0.2221^***^
	(0.0573)		(0.0672)		(0.0240)		(0.0387)
RTETI		−0.0314^***^		−0.0255^***^		−0.0666^***^		−0.0160
	(0.0085)		(0.0092)		(0.0253)		(0.0158)
Individual fixed effect	Yes	Yes	Yes	Yes	Yes	Yes	Yes	Yes
Year fixed effect	Yes	Yes	Yes	Yes	Yes	Yes	Yes	Yes
*R* ^2^	0.5020	0.5398	0.5029	0.5333	0.4698	0.4026	0.5765	0.5944
*N*	14,172	14,172	8,300	8,300	3,688	3,688	2,184	2,184
Hausman test	4,595.22^***^	4,647.55^***^	2,767.46^***^	2,750.43^***^	1,041.42^**^	1,112.94^***^	1,285.58^**^	726.57^***^

^***^p < 0.01, ^**^p < 0.05.

Control variables were added to the model to assess the conditional β convergence. The coefficients of VC technical efficiency in Shanxi and the three regions remained negative and significant at the 1% level, indicating a significant conditional convergence in these regions.

#### 4.2.3 Factors affecting convergence

In Shanxi, the southern region, and the central region, compared to the coefficient of VC technical efficiency in the absolute β convergence analysis, the absolute value of the coefficient of VC technical efficiency in the conditional β convergence analysis increased (albeit it remained negative), demonstrating that the efficiency disparity among VCs narrowed when controlling for influencing factors. The results of the factors that affect the convergence of VC technical efficiency are presented in Table 3.

First, the PS coefficient was not significant in Shanxi or any of the three regions, indicating that PS had no significant effect on the convergence of VC technical efficiency in these areas. Second, the BA coefficient was positive and significant in Shanxi, the southern region, and the central region at the 1% level, indicating that increased BA lowers the convergence of VC technical efficiency in Shanxi, the southern region, and the central region. By contrast, BA had no significant effect on the convergence of VC technical efficiency in the northern region. Third, the PGS coefficient was negative and significant at the 1% level in Shanxi and the southern, central, and northern regions, indicating that government subsidies accelerate the convergence of VC technical efficiency. Finally, the RTETI coefficient was negative and significant at the 1% level in Shanxi, the southern region, and the central region, indicating that the higher the proportion of total expenditure out of the total income of a VC, the higher the convergence of VC technical efficiency.

### 4.3 Endogeneity problem

Endogeneity may be an issue because of the causality between the response variable ln (*TE*_*i, t*+1_/*TE*_*i, t*_) (the average annual growth rate of VC technical efficiency) and the explanatory variable ln *TE*_*i, t*_ (VC technical efficiency in period *t*). The main reason is that VC technical efficiency can affect the average annual growth rate, which in turn can affect VC technical efficiency. To resolve the endogeneity problem, lagged values of the endogenous variables (ln *TE*_*i, t*_) were used as instruments (44), and a two-stage least-square approach was employed (45). In addition, because the explanatory variable (ln *TE*_*i, t*_) was the lag term of the response variable [], the generalized method of moments (GMM) was further used for estimation (46). The results shown in Table 4 indicate that the estimated results are robust to endogeneity problems.

**Table 4:** Endogeneity test of β convergence analyses.

	**Absolut** β **convergence**			**Conditional** β **convergence**		
	**2SLS**	**Different GMM**	**System GMM**	**2SLS**	**Different GMM**	**System GMM**
*TE*_*i, t*_	−0.9176^***^	−0.8644^***^	−0.8586^***^	−0.8690^***^	−0.7709^***^	−0.8107^***^
	764(0.0378)	(0.0532)	(0.0514)	(0.0369)	(0.0423)	(0.2132)
PS				−0.0041	−0.0020	−0.0006
			(0.0054)	(0.0052)	(0.0142)
BA				0.0308^*^	0.1548^*^	0.0990^***^
			(0.0167)	(0.0907)	(0.0380)
PGS				−0.2116^***^	−0.3120^***^	−0.2902^***^
			(0.0098)	(0.0835)	(0.1048)
RTETI				−0.0153^***^	−0.3384^***^	−0.1546^***^
			(0.0043)	(0.1176)	(0.0586)
LM	764.227^***^			759.496^***^		
Wald F	856.372			849.833		
AR(1) (*p*-value)		0.000	0.000		0.000	0.002
AR(2) (*p*-value)		0.371	0.326		0.093	0.543
Hansen test (*p*-value)		0.733	0.167		0.570	0.109

^***^p < 0.01, ^*^p < 0.1.

## 5 Discussion

Since China's new healthcare reform in 2009, the Chinese government has increased investments on health resources for the primary healthcare system in rural areas, including VCs (47). Policymakers must gain a comprehensive understanding of the level of VC technical efficiency in rural areas of China, whether the inequity of VC technical efficiency has diminished over time, and about methods to reduce the disparity of VC efficiency between regions. These insights help in improving the distribution of health resources and health resource utilization efficiency in rural China.

The result of technical efficiency measurement on VCs indicated that VC technical efficiency was low, exhibiting a fluctuating downward trend over time, and there were obvious differences in VC technical efficiency among regions in rural China. These results are supported by Zheng et al. (4), who demonstrated that most provinces of China had inefficient rural medical service systems in 2013–2017 and more than one-third of provinces had lower efficiency scores than the average scores. The low VC technical efficiency can be strongly attributed to the decline in the number of rural doctors and insufficient drug resources. According to the *China Health Statistics Yearbook 2022*, the number of rural doctors decreased from 1,031,828 in 2010 to only 690,561 in 2021, and there have been mass resignations of rural doctors (48). In addition, the decrease in drug expenditure is significantly related to the limited variety of essential medicines because of the Zero-Markup Policy, as reported by Chen et al. (1). As a result, patients prefer to go to higher-level hospitals (49), causing an insufficient number of patients visiting VCs, which leads to poor VC technical efficiency.

This study used convergence analysis models to evaluate the inequity of VC technical efficiency over time. The analysis results of the convergence of VC technical efficiency provided empirical evidence for the absence of α convergence of VC technical efficiency and the occurrence of β convergence in Shanxi and the southern, central, and northern regions over the sample period ( convergence coefficients were negative and significant). Jing et al. (50) reached similar conclusions. In this study, the absolute α convergence results suggested that, without intervention, VC technical efficiency in different regions tends to diverge over time. By contrast, the β convergence results indicated that, when the characteristics of different regions are considered and policy guidance is strengthened, VC technical efficiency in different regions tends to converge. This indicates decrease in the disparity in VC efficiency, signifying the catch-up phenomenon. More importantly, the results of the conditional β convergence analysis demonstrated that catch-up can be improved if factors such as BA, PGS, and internal revenue and expenditure management (based on their relative advantages) of low-efficiency VCs are optimized. This finding is of great significance to the equity of medical services and the rationality of medical resource allocation in rural areas.

In addition, the results of conditional β convergence showed that the main factors that affect convergence were BA, PGS, and RTETI. (1) Increased BA slowed down the convergence of VC technical efficiency in Shanxi, the southern region, and the central region, whereas there was no such effect in the northern region. The coefficient of BA indicated that the average annual growth rate of the efficiency was positively correlated with the level of BA in the base period; the larger the level of BA in the base period, the higher the average annual growth rate of the efficiency. The BA of high-efficiency VCs was larger than that of low-efficiency VCs (51). With the increase in BA, the high-efficiency VCs exhibited a higher average annual growth rate, thus exacerbating the efficiency inequity among VCs. (2) Increased PGS accelerated the convergence of VC technical efficiency and narrowed the disparity in VC technical efficiency. The coefficient of PGS showed that the average annual growth rate of the efficiency was negatively correlated with the level of PGS in the base period, implying that VCs with low PGS in the base period had a higher average annual growth rate. Government subsidies include subsidies for personnel funding, housing, equipment, and implementation of the essential drugs system. High-efficiency VCs receive more PGS in rural China (52). Furthermore, with the increase in PSG, high-efficiency VCs exhibit a low average annual growth rate, whereas low-efficiency VCs exhibit a high average annual growth rate, thus narrowing the efficiency disparity among VCs. A similar result was reported in a previous study (53), which indicated that government health subsidies were progressive and contributed to the narrowing of the gap between poor and rich regions in China. (3) Similarly, increased RTETI narrowed the disparity in VC technical efficiency. The coefficient of RTETI indicated that the average annual growth rate of efficiency was negatively correlated with the level of RTETI in the base period. This implies that VCs with a smaller RTETI in the base period had a higher average annual growth rate than VCs with a large RTETI. As the expenditure of most VCs arises from local government subsidies (54), high-efficiency VCs can receive more subsidies than low-efficiency VCs, leading to a smalle RTETI of low-efficiency VCs. Therefore, government investments can improve the efficiency of low-efficiency VCs more than that of high-efficiency VCs, thus accelerating the convergence and thereby narrowing the disparity in efficiency between high- and low-efficiency VCs (55).

## 6 Conclusion and limitations

### 6.1 Conclusion

This study analyzed the technical efficiency of VCs and its convergence using an efficiency measurement model (SBM) and a convergence model using data from the Shanxi Rural Health Institute 2014–2018 Health Statistics Report. The main conclusions of this study are as follows. First, the VC efficiency from the SBM was low and exhibited obvious differences among regions in rural China. Second there was no α convergence in the VC efficiency, but β convergence occurred in Shanxi and the southern, central, and northern regions over the sample period. Third, the main factors that affect convergence were BA, PGS, and RTETI.

Based on the results, the following policy implications are proposed. First, the government should increase investments in VCs and improve VC technical efficiency by increasing support for rural doctors, including establishing effective promotion mechanisms and providing a higher and more reasonable income (56); improving the Zero-Markup Policy for essential drugs; and increasing drug allocation in VCs (49, 57). Second, the government should be aware of and take measures to curb the inequity in VC technical efficiency among different regions and take suitable measures to curb this disparity. For example, in the southern and central regions, the government should exert moderate control of the scale of VCs, increase government subsidies for VCs, guide VCs to optimize their revenue and expenditure management, and increase PGS regarding low-efficient VCs' expenditure.

### 6.2 Limitations

This study has the following limitations. First, the selection of variables in the VC technical efficiency measurement model and convergence model needs to be improved. Research on VCs is limited, affecting the selection of variables in this study. Future research should focus on using more suitable variables to evaluate VC technical efficiency and convergence. Second, challenges in collecting data over an extended period exist due to factors such as lack of data, statistical data lags, and the impact of the COVID-19 pandemic. The evaluation of VC technical efficiency was restricted to 2014–2018; therefore, future research should extend the research period.

## Data availability statement

The original contributions presented in the study are included in the article/supplementary material, further inquiries can be directed to the corresponding authors.

## Author contributions

YY: Conceptualization, Data curation, Formal analysis, Investigation, Writing—original draft. RE: Conceptualization, Formal analysis, Writing—original draft. XH: Conceptualization, Data curation, Formal analysis, Methodology, Writing—original draft. WX: Conceptualization, Data curation, Formal analysis, Writing—original draft. WL: Conceptualization, Data curation, Formal analysis, Writing—original draft, Writing—review & editing.
